# Evaluation of spine disorders using high contrast imaging of the cartilaginous endplate

**DOI:** 10.3389/fphys.2024.1394189

**Published:** 2024-05-27

**Authors:** Jiyo S. Athertya, Sheronda Statum, Xiaojun Chen, Kevin Du, Soo Hyun Shin, Saeed Jerban, Christine B. Chung, Eric Y. Chang, Yajun Ma

**Affiliations:** ^1^ Department of Radiology, University of California, San Diego, San Diego, CA, United States; ^2^ Radiology Service, VA San Diego Healthcare System, San Diego, CA, United States

**Keywords:** CEP, contrast enhancement, spine imaging, spine disorders, degenerations

## Abstract

**Introduction:** Many spine disorders are caused by disc degeneration or endplate defects. Because nutrients entering the avascular disc are channeled through the cartilaginous endplate (CEP), structural and compositional changes in the CEP may block this solute channel, thereby hindering disc cell function. Therefore, imaging the CEP region is important to improve the diagnostic accuracy of spine disorders.

**Methods:** A clinically available T1-weighted and fat-suppressed spoiled gradient recalled-echo (FS-SPGR) sequence was optimized for high-contrast CEP imaging, which utilizes the short T1 property of the CEP. The FS-SPGR scans with and without breath-hold were performed for comparison on healthy subjects. Then, the FS-SPGR sequence which produced optimal image quality was employed for patient scans. In this study, seven asymptomatic volunteers and eight patients with lower back pain were recruited and scanned on a 3T whole-body MRI scanner. Clinical T2-weighted fast spin-echo (T2w-FSE) and T1-weighted FSE (T1w-FSE) sequences were also scanned for comparison.

**Results:** For the asymptomatic volunteers, the FS-SPGR scans under free breathing conditions with NEX = 4 showed much higher contrast-to-noise ratio values between the CEP and bone marrow fat (BMF) (CNR_CEP-BMF_) (i.e., 7.8 ± 1.6) and between the CEP and nucleus pulposus (NP) (CNR_CEP-NP_) (i.e., 6.1 ± 1.2) compared to free breathing with NEX = 1 (CNR_CEP-BMF_: 4.0 ± 1.1 and CNR_CEP-NP_: 2.5 ± 0.9) and breath-hold condition with NEX = 1 (CNR_CEP-BMF_: 4.2 ± 1.3 and CNR_CEP-NP_: 2.8 ± 1.3). The CEP regions showed bright linear signals with high contrast in the T1-weighted FS-SPGR images in the controls, while irregularities of the CEP were found in the patients.

**Discussion:** We have developed a T1-weighted 3D FS-SPGR sequence to image the CEP that is readily translatable to clinical settings. The proposed sequence can be used to highlight the CEP region and shows promise for the detection of intervertebral disc abnormalities.

## 1 Introduction

Intervertebral disc degeneration (IVD) is a common musculoskeletal disorder, one of the major causes of low back pain (Andersson, 1998). The cartilaginous endplate (CEP) plays a pivotal role as a conduit for nutrient transport, ensuring the nourishment of disc cells which is paramount for sustained disc health (Hwang et al., 2016). The CEP is a thin layer of hyaline-like cartilage situated between the IVD and its adjacent vertebral endplates, predominantly comprised of type II collagen, proteoglycans, and water (Urban et al., 1982; Moon et al., 2013). Because abnormalities of the CEP region have been identified as a potential source of pain (Fields et al., 2014). Thus, there is a need to evaluate the CEP region for a comprehensive assessment of low back pain.

Magnetic Resonance Imaging (MRI) is a non-invasive and non-ionizing tool for diagnosing various spinal disorders in clinical routine (Cousins and Haughton, 2009). Clinical MRI has concentrated primarily on the nucleus pulposus (NP) and annulus fibrosis (AF) to evaluate disc degeneration, with limited access to the CEP. This is because the T1- and T2- weighted fast-spin-echo (T1w-FSE and T2-FSE) sequences used for clinical evaluation have relatively long echo times which cannot capture the fast decaying signals of the short T2 CEP efficiently.

Ultrashort echo time (UTE) sequences, with echo times less than 100 µs, have been successfully applied for CEP imaging (Afsahi et al., 2021; Ma et al., 2022). Recently, several UTE-based techniques have been developed for selective imaging of the short T2 CEP using either subtraction or inversion-based approaches (Bae et al., 2013; Cai et al., 2020; Lombardi et al., 2021a; Lombardi et al., 2021b; Athertya et al., 2023; Finkenstaedt et al., 2023). Various quantitative UTE biomarkers have also been identified to have various relationships with CEP dysfunction and/or IVD degeneration (Fields et al., 2015; Wang et al., 2021; Wei et al., 2022). However, these UTE sequences have mainly been used for research purposes, and not all MRI vendors offer UTE sequences that are easily accessible for routine clinical use. Moreover, the scan time of these UTE-type protocols is relatively long for clinical implementation, limiting the usage of UTE sequences in large cohort studies. Many studies have therefore focused their investigations solely on cadaveric specimen imaging (Bae et al., 2013; Berg-Johansen et al., 2018).

As reported in previous studies, the T2 and T2* values of the CEP are ∼18 ms and ∼15 ms, respectively (Wang et al., 2021; Athertya et al., 2022) Thus, it is possible to detect CEP signals using gradient recalled-echo sequences with minimized echo times (e.g., 2.2 ms), which have been validated in previous sample studies (Disler et al., 1994; Moon et al., 2013).

In this study, we propose to optimize a T1-weighted and fat-suppressed 3D spoiled gradient recalled-echo (3D FS-SPGR) for high-contrast CEP imaging *in vivo*. The FS-SPGR sequence is clinically available with high scan efficiency. A relatively high flip angle (FA) is employed to produce T1 weighting in the FS-SPGR sequence to improve the contrast between the short T1 CEP and the long T1 NP. The fat suppression in the FS-SPGR sequence further enhances the CEP contrast relative to the marrow fat. In this study, seven asymptomatic volunteers and eight patients with lower back pain were recruited and scanned on a 3T whole-body MRI scanner to investigate the technical feasibility of high-contrast CEP imaging using the optimized FS-SPGR sequence. The clinical T1w-FSE and T2-FSE sequences were also scanned for comparison.

## 2 Materials and methods

### 2.1 MR acquisition sequence

The proposed study was approved by the institutional review board. All sequences were implemented on a 3T GE MR750 clinical scanner (GE Healthcare Technologies, Milwaukee, Wisconsin). A four-channel phased array spine coil was utilized for signal reception.

**FIGURE 1:**
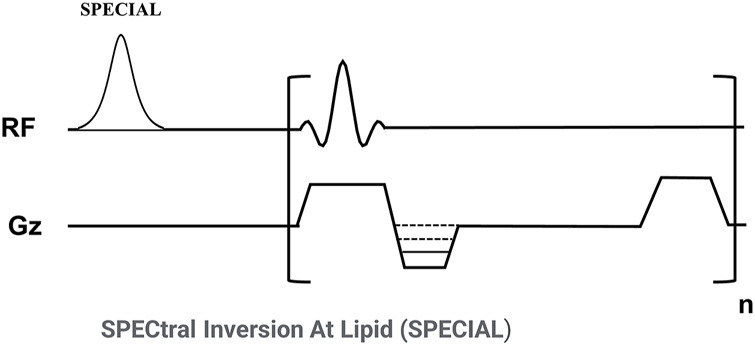
Sequence diagram of the 3D fat-suppressed spoiled gradient echo (FS-SPGR) sequence. The product SPECtral Inversion At Lipid (SPECIAL) technique was used for fat suppression.

Figure 1 shows the sequence diagram of the 3D FS-SPGR which has been routinely used for clinical liver imaging [also known as Liver Acceleration Volume Acquisition (LAVA)] (Rofsky et al., 1999). The product SPECtral Inversion At Lipid (SPECIAL) technique was used for fat suppression (Del Grande et al., 2014).

Since the CEP has a much shorter T1 than NP for a 3T clinical scanner (∼540 ms vs. ∼1,250 ms) (Moon et al., 2013), a strong T1 contrast can be utilized to highlight the CEP signals (Lombardi et al., 2021b). A high FA and a relatively short TR are employed in the FS-SPGR sequence to produce high T1 contrast. A minimum TE was used to capture sufficient signals from the short T2 CEP (T2* of ∼15 ms) (Wang et al., 2021). Routinely used clinical spine imaging sequences including chemical fat-suppressed (FS) T2w-FSE and non-FS T1w-FSE techniques were also scanned for comparison.

### 2.2 *In vivo* human spine imaging

Seven asymptomatic controls (mean age: 30 ± 4 years old, 38% female) and eight patients with lower back pain (mean age: 58 ± 12 years old, 43% female) were recruited for this study. Written informed consent was obtained from each participant before the imaging examinations.

The inclusion criteria included adults aged between 18 and 90 years old, without low back pain or degenerative disc diseases for healthy controls, and with low back pain for patients. The definition criteria for healthy subjects were as follows: no history of diagnosed intervertebral disc degeneration and no functional impairment or moderate to severe physical symptoms in the past 6 months in the spinal region. The definition criteria for low back pain patients was defined as per NIH reports (Deyo et al., 2014). We acknowledge that the definition of chronic low back pain by the NIH Task Force describes it as pain persisting for half the days or more over a 6-month period. Exclusion criteria included presence of a metal implant, claustrophobia or any other contraindication to MRI, prior or planned spinal fusion surgery, as well as current or planned pregnancy for female subjects.

In the context of healthy control imaging, the FS-SPGR sequence was conducted three times, encompassing a single breath-hold scan and two free-breathing scans. The individuals remained inside the scanner and maintained consistent positioning throughout the various acquisitions. The number of excitations (NEX) signifies how many times each line of k-space data is acquired for signal averaging, commonly employed to enhance the image SNR. The sequence parameters remained consistent, except that one of the free-breathing scans was executed with an NEX of 4 to enhance signal-to-noise ratio (SNR) performance. For the patient scans, only the free-breathing scan with an NEX of 4 was performed, as it yielded the highest image quality (see results below). The parameters of all the sequences are provided in Table 1.

**TABLE 1:** Sequence parameters for the *in vivo* spine studies.

Parameters	T2w-FSE	T1w-FSE	FS-SPGR, NEX = 1 w/BH	FS-SPGR, NEX = 1 w/o BH	FS-SPGR, NEX = 4 w/o BH
Voxel size (mm^3^)	0.9 × 1.3 × 3	1.1 × 1.3 × 3	0.9 × 1.3 × 3	0.9 × 1.3 × 3	0.9 × 1.3 × 3
Matrix	360 × 270	300 × 260	300 × 210	300 × 210	300 × 210
TR (ms)	4,019	692	5.1	5.1	5.1
TE (ms)	102	42	2	2	2
FA (°)	142 (Refocus)	111 (Refocus)	25	25	25
No. of slices	24	24	36	36	36
BW (kHz)	50	50	83.33	83.33	83.33
Scan time	2 min 33 s	2 min 45 s	24 s	24 s	1 min 36 s

BH, breath-hold.

### 2.3 Data analysis

To compare the CEP contrast among the three FS-SPGR scans in asymptomatic volunteers, the CNRs between CEP and NP (CNR_CEP-NP_) and between CEP and BMF (CNR_CEP-BMF_) were computed. These CNRs were determined as the differences in mean signal intensities between these tissues divided by the background noise. The background noise was estimated as the standard deviation of signals measured within a region of interest (ROI) (∼6 × 6 cm^2^) in an artifact-free area.

## 3 Results

**FIGURE 2:**
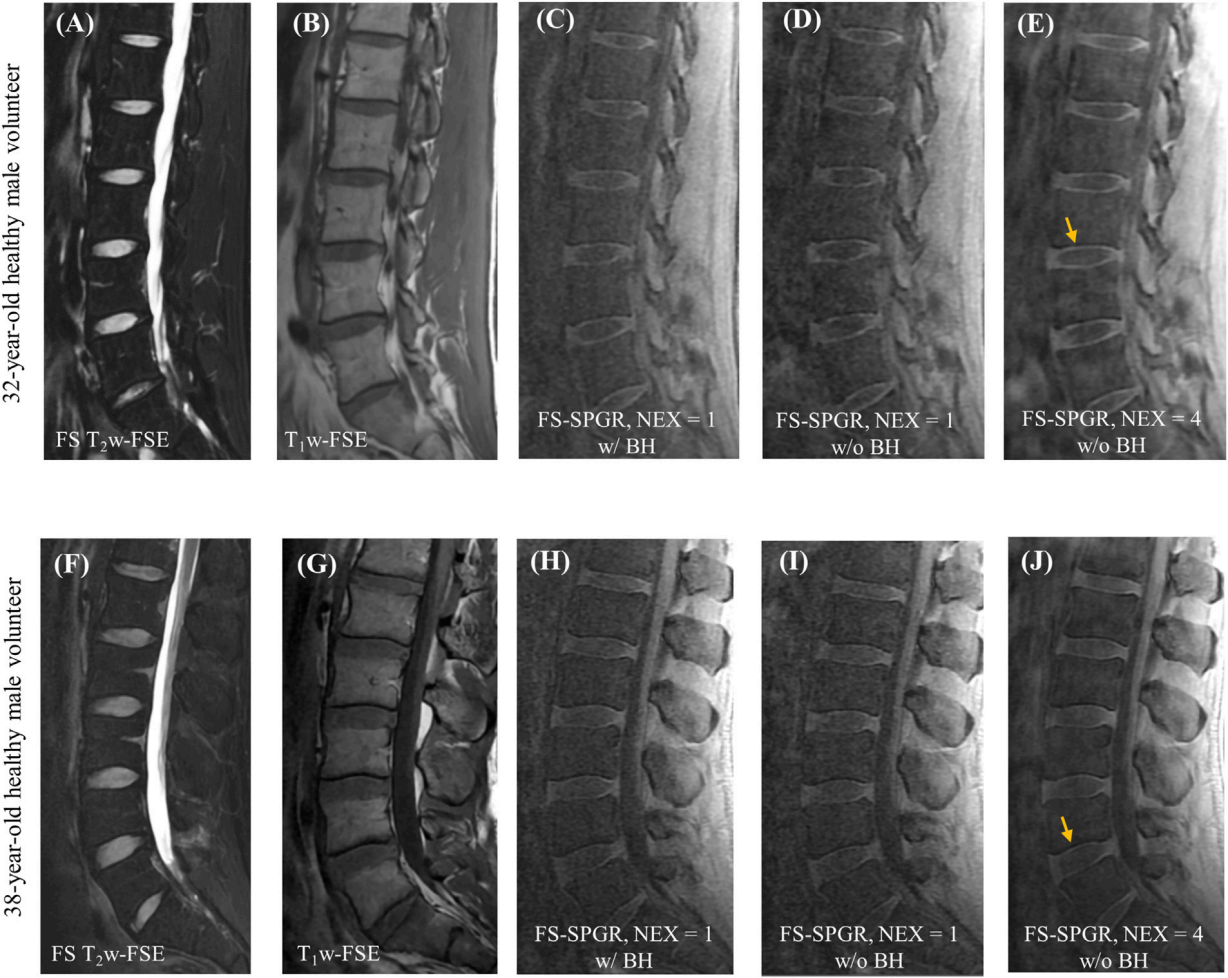
Clinical FS T_2_w-FSE **(A,F)** and T_1_w-FSE **(B,G)** images as well as 3D FS-SPGR images **(C–E,H–J)** from a 32-year-old and a 38-year-old healthy male subject, respectively. Panels **(C,H)** and **(D,I)** show the single NEX FS-SPGR images with and without breath-hold (BH) for the two different controls respectively. To improve the SNR, NEX was increased to 4 for the free breathing scan **(E,J)**, with yellow arrows indicating the high contrast CEP region.

Figure 2 shows representative lumbar spine images acquired using the 3D FS-SPGR sequence from two different asymptomatic control subjects. The FS T2w-FSE and non-FS T1w-FSE images were unable to highlight the CEP signals due to their relatively long TE. The CEP regions were visually accentuated in the FS-SPGR images obtained with and without breath-hold. It is worth noting that although the breath-hold scan provided a sharper background compared to the free-breathing scan with NEX of 1, its SNR was relatively low. To improve SNR performance, NEX was increased to 4 for the free-breathing scans, yielding the highest quality CEP imaging.

Table 2 provides a summary of mean CNR_CEP-NP_ and CNR_CEP-BMF_ measurements for the three FS-SPGR scans, i.e., under free breathing conditions at NEX = 1 and NEX = 4 as well as with breath-hold conditions, for seven asymptomatic volunteers. The CNR measurements of the patient group are also included for comparison. Of all the scans for healthy volunteer group, the highest contrast for CEP vs. NP (6.1 ± 1.2) and CEP vs. BMF (7.8 ± 1.6) was achieved at NEX = 4 under free breathing conditions, followed by breath-hold, and under free breathing with NEX = 1. Conversely, negative CNR_CEP-NP_ and CNR_CEP-BMF_ values were obtained for the abnormal CEPs in patients with LBP. Significant difference of the CEP CNR measurements were found between normal and patient cohorts for the scans with NEX = 4 under free breathing condition (*p* < 0.0001).

**TABLE 2:** Summary of the mean CNR_CEP-NP_ and CNR_CEP-BMF_ measurements from the FS-SPGR scans for seven asymptomatic volunteers and eight low back pain patients.

CNR	FS-SPGR, NEX = 1 w/BH (Control)	FS-SPGR, NEX = 1 w/o BH (Control)	FS-SPGR, NEX = 4 w/o BH (Control)	FS-SPGR, NEX = 4 w/o BH (patient)
Between CEP and NP	2.8 ± 1.3	2.5 ± 0.9	6.1 ± 1.2	−8.2 ± 7.4
Between CEP and BMF	4.2 ± 1.3	4.0 ± 1.1	7.8 ± 1.6	−0.1 ± 4.2

BH, breath-hold; BMF, bone marrow fat; NP, nucleus pulposus.

**FIGURE 3:**
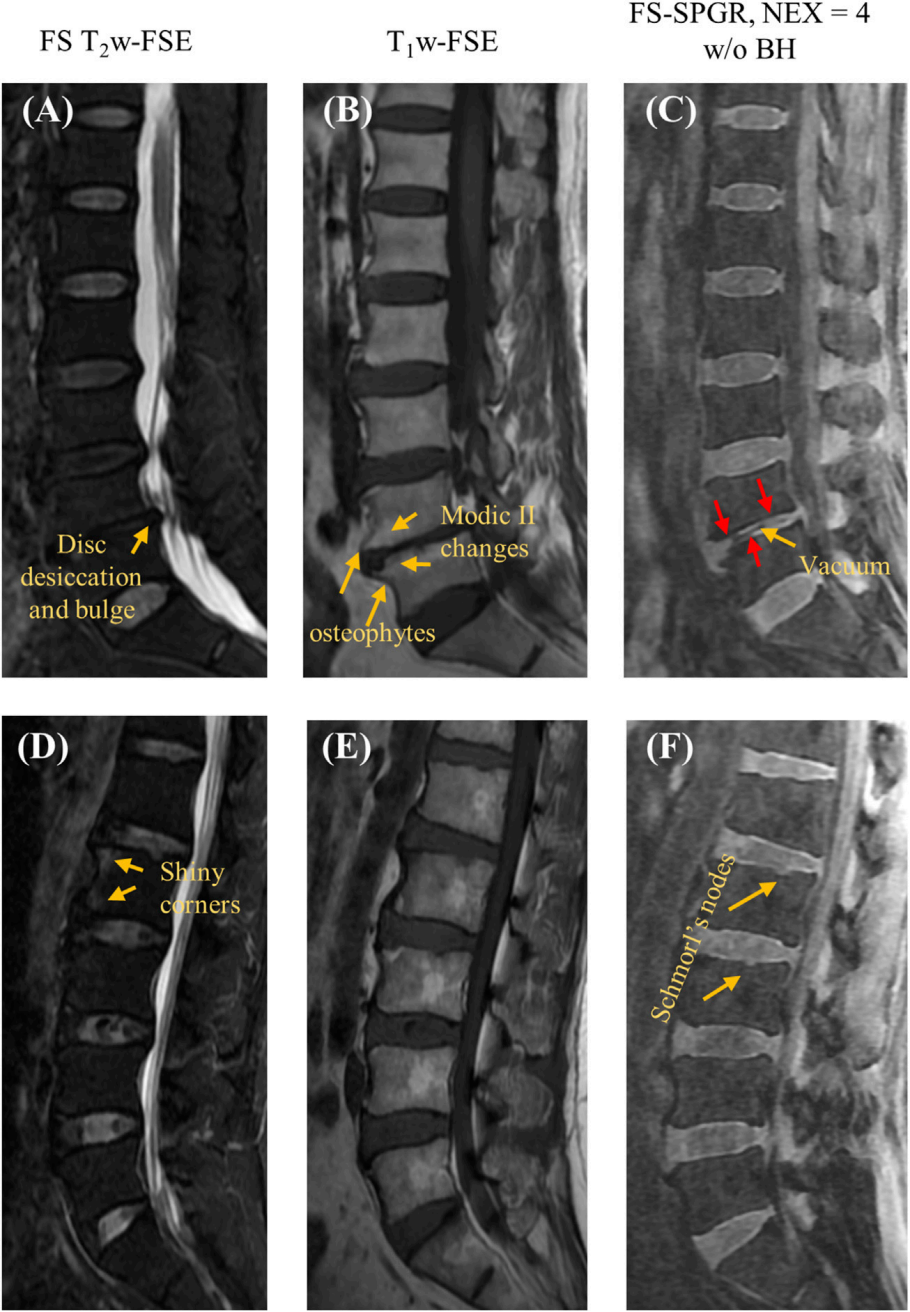
Clinical FS T_2_w-FSE **(A,D)** and T_1_w-FSE **(B,E)** images as well as 3D FS-SPGR (free breathing, NEX = 4) **(C,F)** images acquired from two patients with lower back pain (first row: 67-year-old female; second row: 61-year-old male). The abnormal CEP regions are indicated by red arrows in panels **(C,F)** while other pathologies like disc desiccation [panel **(A)**], Schmorl’s nodes [panel **(F)**], Modic II changes [panel **(B)**] are pointed by yellow arrows.

Figure 3 shows the FS-SPGR images (free breathing, NEX = 4) along with clinical images acquired from two different patients (67 years old, female and 61 years old, male, respectively) with lower back pain. For the first patient, Figures 3A–C exhibit degenerative disc disease at L4-5, marked by disc bulging, desiccation, vacuum phenomenon, fatty marrow replacement (Modic II changes), and marginal osteophytes. The arrows indicate areas of CEP loss, greatest at the anterosuperior aspect of L5. For the second patient, Figures 3D–F reveal shiny corner lesions that are most prominent at the anterosuperior and anteroinferior aspects of the L2 vertebral body. The proposed FS-SPGR sequence depicts small Schmorl’s nodes from L1 through L3, while the superior L1 nodes have preserved CEPs. In contrast the superior L2 and L3 levels exhibit disrupted CEPs covering the nodes.

**FIGURE 4:**
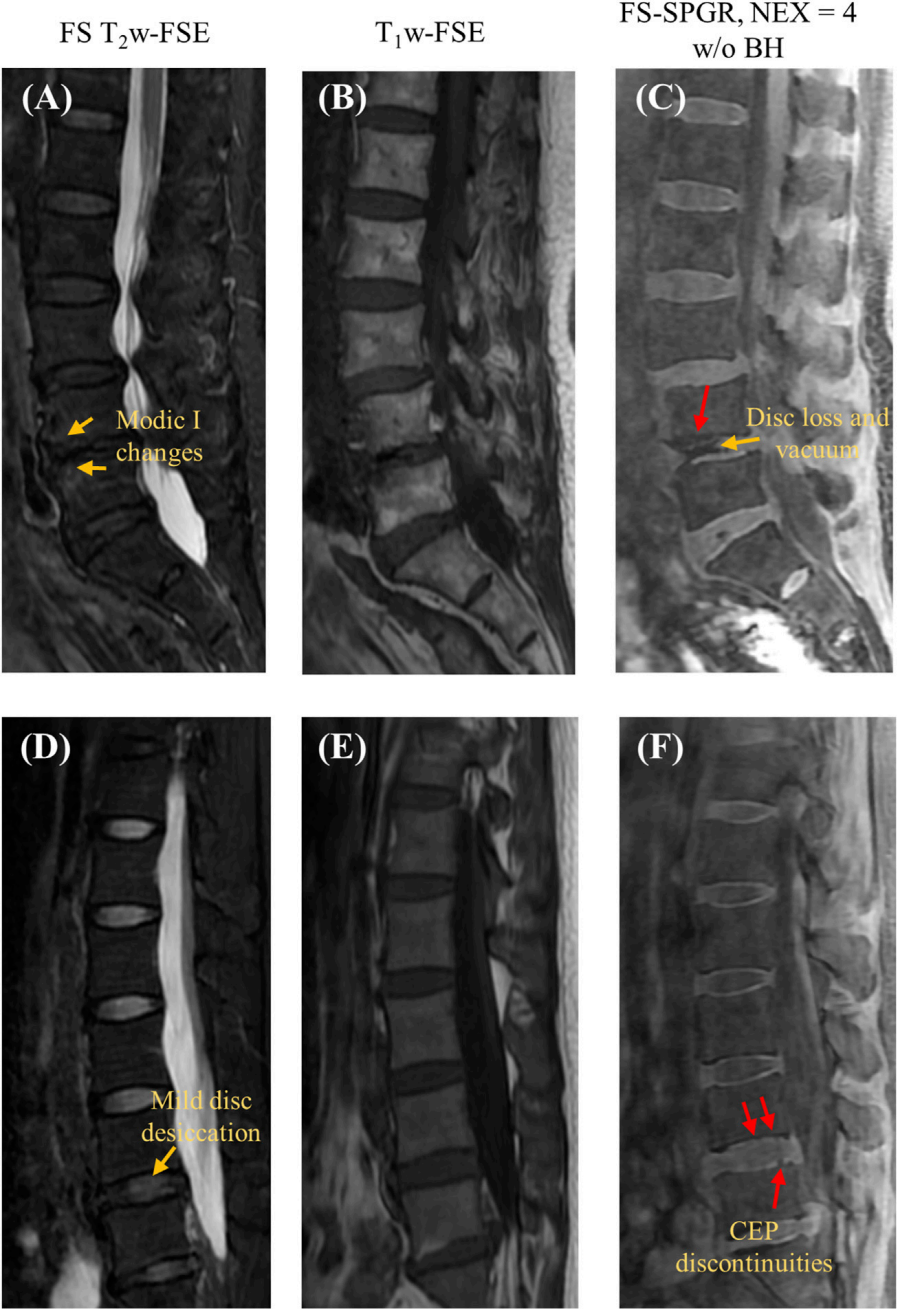
Clinical FS T_2_w-FSE **(A,D)** and T_1_w-FSE **(B,E)** images as well as 3D FS-SPGR (free breathing, NEX = 4) **(C,F)** images acquired from two patients with lower back pain (first row: 70-year-old female; second row: 46-year-old female). The CEP discontinuities are indicated by red arrows in panels **(C,F)** while the other pathologies, namely, Modic I changes [panel **(A)**], mild disc desiccation [panel **(C)**] and disc loss and vacuum [panel **(C)**] are marked by yellow arrows.

Figure 4 shows the FS-SPGR images alongside clinical images obtained from two female patients (70 and 46 years old, respectively) with lower back pain. For Figures 4A–C, the degenerative disc disease is evident at L4-5, characterized by disc loss, vacuum phenomenon, and anterior fibrovascular marrow changes (Modic I) and osteophytes. The CEP regions at this level are both grossly abnormal, though with different appearances. The inferior L4 CEP displays a hypointense appearance, consistent with a change in composition, while the superior L5 CEP is mildly hyperintense and significantly thickened. Both changes are consistent with the CEP dysfunction. For Figures 4D–F, there is mild disc desiccation at the L4-5 with associated subtle changes of the CEPs (arrows point to foci of hyperintensity consistent with compositional change). Additionally, mild disc desiccation is observed at the L5-S1 level, featuring a small area of anterior fatty marrow replacement (Modic II change). The anterior half of the L5-S1 CEPs appear hypointense, consistent with compositional alterations.

**FIGURE 5:**
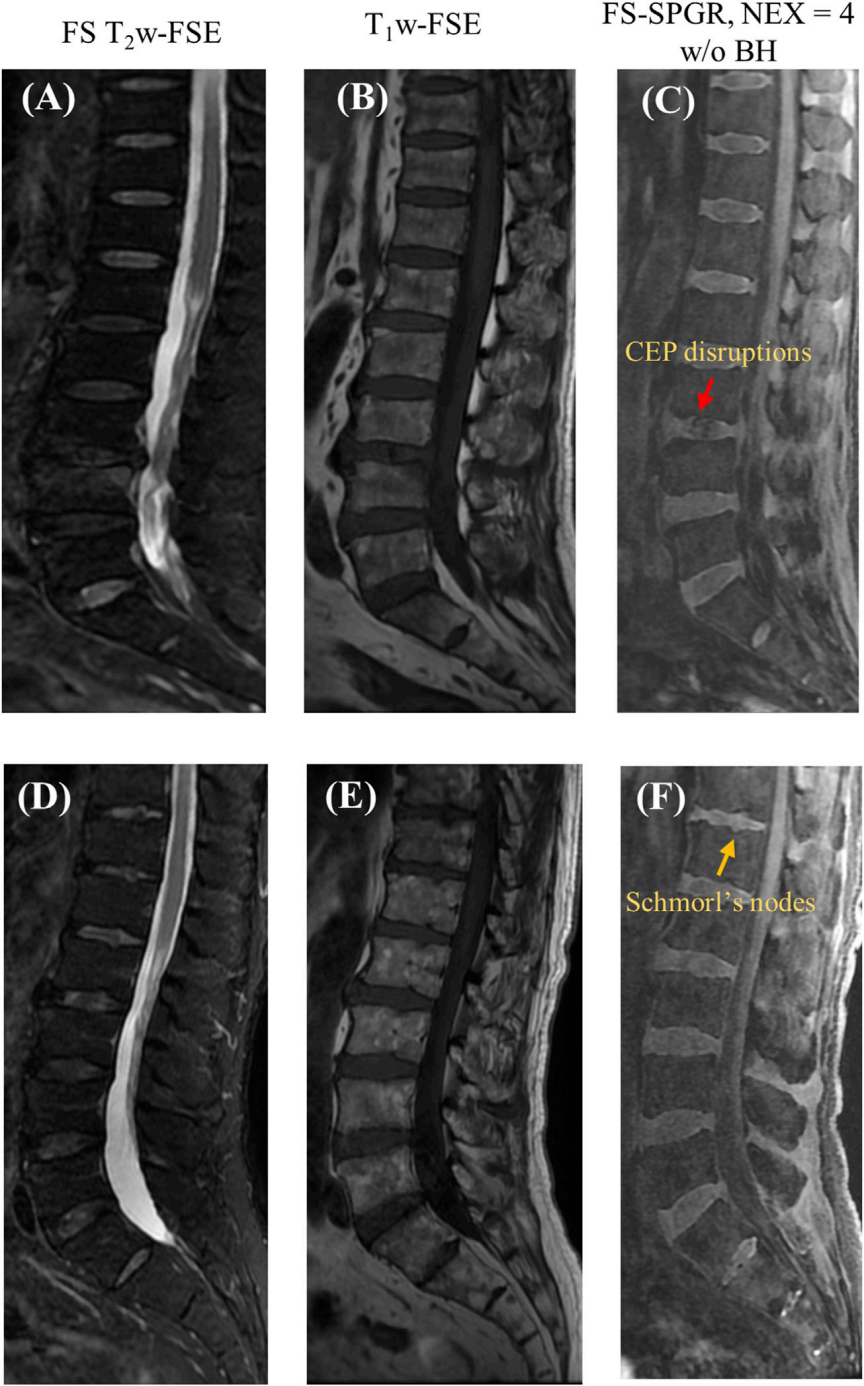
Clinical FS T_2_w-FSE **(A,D)** and T_1_w-FSE **(B,E)** images as well as 3D FS-SPGR (free breathing, NEX = 4) **(C,F)** images acquired from two patients with lower back pain (first row: 63-year-old male; second row: 67-year-old male). The CEP disruptions are pointed by red arrow and Schmorl’s nodes are indicated by yellow arrows in panels **(C,F)**, respectively.

Figure 5 presents two rows of the FS-SPGR images alongside the routine clinical sequences for another two male subjects (63 and 67 years old, respectively) with lower back pain. The first row shows a longer segment of CEP disruption at this level of degenerative disc disease, while the second row shows visibly delineated Schmorl’s nodes.

Similar to the control study, clinical sequences failed to capture signals from the CEP for all the patients due to the region’s relatively long TE, thus cannot be utilized for CEP evaluation. In contrast, the 3D FS-SPGR sequence distinctly delineated the CEP regions from the vertebral body and intervertebral disc. These findings demonstrate that the optimized 3D FS-SPGR sequence can emphasize the CEP region and detect CEP abnormalities in patients, showing promise for clinical diagnosis of low back pain.

## 4 Discussion

In this work, we proposed a highly T1-weighted 3D FS-SPGR sequence for high-contrast lumbar spine CEP imaging. The strong T1 weighting in the FS-SPGR sequence improved the contrast between the short T1 CEP and the long T1 NP, and the SPECIAL fat suppression technique further enhanced the CEP contrast relative to marrow fat. With this optimized FS-SPGR technique, distinct continuous lines indicative of signal from the CEP were identified in healthy subjects whereas abnormal IVDs in the patient population exhibited discontinuity or irregularities in the CEP. Thus, this time-efficient and clinically available FS-SPGR sequence has great potential to be used for studying low back pain with a large cohort in future.

Many studies have indicated that structural and compositional changes of the CEP contribute to the onset and progression of degenerative disc diseases (Boyd and Carter, 2006; Rajasekaran et al., 2008; Cao et al., 2020). A dedicated examination of CEP degeneration holds significance for elucidating the mechanisms underlying disc degeneration. This knowledge could be crucial for the prevention and diagnosis of various spinal degenerations at an early stage.

Imaging of the CEP using conventional FSE-type sequences is challenging due to CEP’s diminutive dimensions and relatively short T2/T2* tissue characteristics which renders the CEP nearly imperceptible and indistinguishable from the adjacent bone and soft tissues. While the routinely used spine MRI (i.e., the FSE-type sequences) allow for gross morphologic evaluation of the IVD, the sequences fail to capture both the integrity of the CEP and visualization of its fine anatomic structures.

The T1-weighted FS-SPGR sequence with a minimal TE proposed in this study provides high contrast CEP imaging, demonstrating that the clinical available SPGR sequence is able to capture signals from the CEP, which has a relatively short T2 relaxation time. In comparison to the free breathing scans, 24-s breath-hold scans show fewer motion-related artifacts, but with limited SNR. That being said, some patients may not be able to hold their breath for 24 s, lessening this approach’s practical usefulness. By comparison, the free breathing scan with NEX = 4 has a higher CNR and is particularly advantageous for individuals with breath-holding difficulties.

Despite being preliminary and based on a small sample size, the findings of this study suggest that distinctive abnormalities in CEP morphology, including Schmorl’s nodes and disruptions in the CEP, are potentially associated with disc level and overall disc health. Quantitative assessment of pathological changes of CEP is important for diagnosis, especially for the longitudinal studies to test effects of potential therapies. One approach for quantification is through the comprehensive analysis of CEP morphology, namely, the thickness, signal intensity, and CEP to disc ratio. Nevertheless, further investigation with a larger and more diverse sample encompassing various degeneration levels will be essential to validate these correlations.

UTE sequences are known for their ability to image tissues with short T2/T2* on the order of a few hundred microseconds, short enough to sufficiently highlight the CEP region (Chang et al., 2015). However, in the current stage of their development, center-out UTE sequences often suffer from eddy currents-induced artifacts, relegating these sequences mainly to the realm of research. Further, most clinical scanners have limited access to more advanced UTE sequences, which limits their usage in CEP imaging as part of clinical routine. The scan time of the IR-based UTE sequence is also relatively long for the clinical setting. Fortunately, because the FS-SPGR sequence is the inbuilt vendor sequence for clinical MR equipment, the proposed FS-SPGR sequence has great potential to facilitate smooth clinical translation of the CEP imaging.

There are a few limitations in this study. First, the limited sample size prevented us from investigating the relationship between CEP abnormalities and degenerative changes in the IVD. Second, while the CNR is still limited in comparison to UTE-based techniques, it has the potential for further enhancement through the application of deep learning-based denoising techniques. For instance, with an effective denoising process, the NEX can be reduced to improve the scan efficiency while maintaining sufficient SNR performance.

## 5 Conclusion

The commercially accessible 3D FS-SPGR sequence is proficient at generating high-contrast imaging of the CEP for the evaluation of spinal disorders. FS-SPGR can effectively discern CEP abnormalities that remain imperceptible in standard clinical FS T2w-FSE and non-FS T1w-FSE sequences. Given the relatively short *in vivo* scanning duration, this technique will offer novel means of non-invasive evaluation and measurement of spinal health.

## Data Availability

The raw data supporting the conclusion of this article will be made available by the authors, without undue reservation.
